# The Reversal Characteristics of GABAergic Neurons: A Neurovascular Model

**DOI:** 10.1115/1.4056336

**Published:** 2022-11-29

**Authors:** Tim David, Robin Morillo, Clare Howarth, Jason Berwick, Llywelyn Lee

**Affiliations:** Department of Mechanical Engineering University of Canterbury Christchurch, New Zealand; Department of Mathematics North Carolina State University; Department of Psychology University of Sheffield, U.K; Department of Psychology University of Sheffield, U.K; Department of Psychology University of Sheffield, U.K

## Abstract

Neurovascular coupling (NVC) is the ability to locally adjust vascular resistance as a function of neuronal activity. Recent experiments have illustrated that NVC is partially independent of metabolic signals. In addition Nitric Oxide (NO) has been shown in some instances to provide an important mechanism in altering vascular resistance. An extension to the original model of NVC [1] has been developed to include the activation of both somato-sensory neurons and GABAergic interneurons and to investigate the role of NO and the delicate balance of GABA and neuronal peptide enzymes (NPY) pathways. The numerical model is compared to murine experimental data that provides time-dependent profiles of oxy, de-oxy and total-haemoglobin. The results indicate a delicate balance that exists between GABA and NPY when n-NOS interneurons are activated mediated by NO. Whereas somato-sensory neurons (producing potassium into the extracellular space) do not seem to be effected by the inhibition of NO. Further work will need to be done to investigate the role of NO when stimulation periods are increased substantiallyfrom the short pulses of 2 seconds as used in the above experiments.

## 1 Introduction

Neurovascular coupling (NVC) is the ability to **locally** adjust vascular resistance as a function of neuronal activity and is believed to be mediated by a number of different signalling mechanisms. It allows the increase of oxygen and glucose to be transported to activated neurons since they cannot support the storage of nutrients. Recent experiments illustrated that the NVC response is partially independent of metabolic signals such as CO_2_ [2, 3, 4, 5, 6]. An alternative to the original metabolic theory of [7] was proposed where the neuron releases signalling molecules to directly or indirectly affect the blood flow. Many mechanisms such as the K^+^signalling mechanism [8], the nitric oxide NO (NO) signalling mechanism or the arachidonic acid (AA) to Epoxyeicosatrienoic acid EET (EET) pathway are found to contribute to the neurovascular response [9].

The K^+^signalling mechanism of NVC seems to be supported by significant evidence, although new evidence shows that the endfoot astrocytic Ca^2+^ could play a significant role. The K^+^signalling hypothesis mainly utilises the astrocyte, positioned to enable the communication between the neurons and the local perfusing blood vessels. The astrocyte and the endothelial cell (EC) surrounding the perfusing vessel lumen exhibit a striking similarity in ion channel expression and thus can enable control of the smooth muscle cell (SMC) from both the neuronal and blood vessel components [10]. Whenever there is neuronal activation K^+^ ions are released into the extra-cellular space and synaptic cleft. The astrocyte is depolarised by taking up K^+^released by the neuron and releases it into the perivascular space via the endfeet through the K^+^BK channels [11]. This increase in extra cellular space K^+^concentration (3 – 10 mM) near the arteriole hyperpolarises the SMC through the inwardly rectifying K^+^channel (KIR channel), effectively closing the voltage-gated Ca^2+^channel, reducing smooth muscle cytosolic Ca^2+^and thereby causing dilation. To initiate dilation/contraction of the vessel our full model uses the experimental data of Filosa et al [8] where with increasing potassium concentration in the perivascular space the Nernst potential moves to the right this is modelled using a function of perivascular [K+]. For the BK channels we utilise a conductance that is a function of [Ca2+], membrane voltage (changing as a function of [K+]) and 20-HETE see [12, 13]. An existing neuron model [14, 15] has been extended to include an additional transient Na^+^ion channel (NaT) expressed in the neuron, and integrated into a complex NVC model [12, 13]. This present model is based on the hypothesis that the K^+^signalling mechanism of NVC is the primary contributor to the vascular response and the Na^+^K^+^exchange pump in the neuron is the primary consumer of oxygen during neural activation. The model contains more than 250 parameters, most of which come from non-human experiments. In the interests of space a full description of the model and its assumptions can be found in [1].

Lately there has been some discussion about the role of NO in NVC and its importance. [1] showed that the K^+^pathway governs the fast onset of vasodilation while the NO pathway has a delayed response [16].

In the majority of cases excitatory neurons produce, on the whole, glutamate as a neurotransmitter as well as neuronal derived NO. Glutamate is the first molecule in the AA pathway culminating in the production of 20-Hydroxyeicosatetraenoic acid (20-HETE) which ultimately inhibits the K^+^channels in the SMC. Whereas inhibitory (GABAergic) neurons of certain types produce γ-Aminobutyric acid (GABA) and neuropeptide Y (NPY). These two pathways have contrasting effects. NPY mediates the opening of the voltage operated calcium channels (VOCC) in the SMC, producing a constriction in the arterioles whilst GABA mediates the Cl^-^channels hyperpolarising the SMC and allows the arterioles to dilate. In this case NO seems to play an important role since for normal NVC to occur, when inhibitory neurons are activated, there is a balance between NPY and GABA mediated pathways. The reason is due to two conditions. Firstly NO inhibits the catalysing of GABA (via GABA-T, a particulate enzyme that is present in high concentration in GABAergic neurons.) to glutamate. Secondly NO also inhibits the production of 20-HETE in the SMC. There is evidence to suggest that in patients with Alzheimer’s disease NO has a lower concentration than normal [17]. The question now arises whether the cessation of the production of NO during neuronal activation causes an imbalance between the competing effects of NPY and GABA and disrupts the flow of nutrients to activated neurons, a crucial aspect in neurological disease. We have developed an extension to the original model of NVC [1] to investigate the role of NO and the delicate balance of GABA and NPY pathways.

**Fig. 1:**
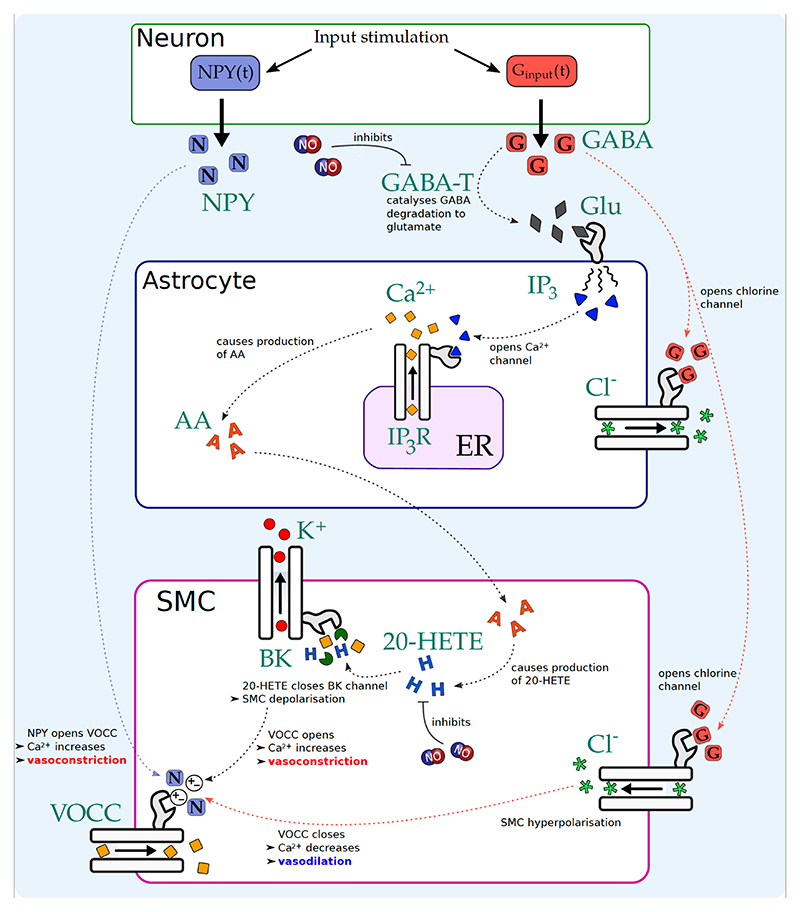
Schematic diagram of the GABA and NPY pathways in an interneuron model. Neuronal stimulation causes a release of NPY and GABA from the neuron. NPY opens the VOCC causing vasoconstriction. GABA opens Cl^-^channels on the SMC causing cell hyperpolarisation and hence closes the VOCC leading to vasodilation. GABA also degrades to glutamate leading to an increase in astrocytic Ca^2+^and AA. AA diffuses to the SMC and increases 20-HETE concentration which closes the BBK channel channel. This leads to cell depolarisation, opening the VOCC and leading to vasoconstriction.

The parameter values for the full model (excluding the GABA/NPY components) were obtained through a two part sensitivity analysis and optimization routine. First through Monte Carlo sampling we were able to identify a subset of the parameters for which altering their values had significant impact upon the model’s accuracy to the experimental data ([18]). Then working with this much smaller set of parameters we minimized the discrepancy between the model’s behaviour and the experimental data. In total 40 parameters were changed, the largest change was 14% (astrocytic Na+ channel conductance), the next largest at 10% (rate of astrocytic IP3 production due to glutamate receptors,) the average amount changed was only 1.1%. Figure 1 shows a sketch of the GABA /NPY pathways.

## 2 Experiments

Experimental data are taken from experiments performed by Lee et al. [18], in which full experimental details can be found. In brief, stimulation-evoked cortical haemodynamic responses were measured using 2-Dimensional Optical Imaging Spectroscopy (2D-OIS: [19]), which allows the measurement of relative changes in oxyhaemoglobin (HbO), deoxyhaemoglobin (HbR) and total haemoglobin (HbT). Specific activation of nNOS-expressing interneurons was achieved through the use of an optogenetic approach.Experiments used lightly anaesthetised nNOS-CreERT x ChR2-eYFP mice (12 mice; 6 male, 6 female), aged 16-36 weeks old and weighing 20-36g. Haemodynamic responses to stimulation were assessed before and after systemic injection of N(ω)-nitro-L-arginine methyl ester (LNAME), a non-selective NOS inhibitor; 75mg/kg, i.p., which has previously been shown to reduce cortical NOS activity by over 90 % [20]. Data were taken from an ROI indicating the right whisker barrel cortex.

### 2.1 Whisker/Optogenetic stimulation

To drive neural activity and associated haemodynamic responses in the right hand whisker barrel cortex, whiskers on the left hand side were stimulated (2s, 5Hz) using a plastic T-bar attached to a stepper motor. For the optogenetic stimulation a fibre-coupled 470 nm LED light source was placed above the skull, in the centre of the right whisker barrel cortex. 2s periods of photostimulation (99Hz, 10ms, 1V, 0.45mW) were used to activate ChR2, activating nNOS-expressing interneurons within the cortex. Whisker and optogenetic stimulations were interleaved with an ISI of 25s. 20-30 repeats of each stimulation type were acquired per animal.

## 3 Modelling

### 3.1 Interneuron Model

The mathematical derivation of the model can be understood with reference to Figure 1. The time-dependent GABA concentration is determined with an o.d.e (see equation 1) that is comprised of a degradation term (proportional to GABA-T activity) and a time dependent input simulating GABA release from the neuron during neuronal stimulation. The GABA-T activity is modelled as a sigmoid function that decreases with neuronal NO concentration.

The glutamate concentration is determined with an o.d.e (see equation 5) and increases either due to vesicle release from an excitatory neuron or due to the degradation of GABA in an inhibitory neuron.

Two GABA dependent Cl^-^channels are added, with one on the astrocyte and one on the SMC. These channels have a reversal potential of −75 mV and a channel conductance dependent on GABA; when GABA = 0, conductance = 0 and when GABA is at some maximal value, the conductance is also some maximal value *G_GABA_*. At present this maximum is taken as ***G_GABA_*** = 0.3 × ***G_cl,i_*** where ***G_cl,i_*** is the conductance of the SMC Cl^-^leak channel (value chosen to fit with Lee et al. [18] experimental data).

The conductance of the VOCC in the SMC is multiplied by an NPY dependent function such that when NPY= 0, the VOCC conductance *g_VOCC_* is equal to the regular conductance *G_Caj_* of the VOCC, and when NPY is at some maximal value *g_VOCC_* is equal to 1.05 × *G_Ca_,_i_* (value chosen to fit with the Lee et al. [18] experimental data).

The equations for the model are given below with associated parameters found in Table 1.

### 3.2 GABA

The model parameters used in the following equations are chosen to fit with the experimental findings of [21] and [22] so that when LNAME is administered we obtain a decrease of 50% in GABA concentration and increase of 100% in GABA-Tactivity.

The GABA concentration is nondimensionalised with respect to some maximal value so that at rest the concentration is zero and during regular neuronal stimulation the nondimensional concentration *GABA_N_* is 1. A simple o.d.e for *GABA_N_* including production and degradation is then written as

(1) $$\frac{dGABA_{N}}{dt}=-\kappa_{GABA}GABA_{N}+\alpha_{GABA}I_{H}(t)$$

where the first term on the RHS models GABA degradation and *I_H_ (t)* is a time dependent input function simulating an input of GABA during neuronal stimulation modelled via a heaviside function:

(2) $$I_{H}(t)=\{\begin{array}{ll} 1 & t_{0}<t<t_{0}+\Delta t\\ 0 & \mathrm{otherwise} \end{array}$$

where *t_0_* is the beginning of stimulation and △t is the length of stimulation. The rate at which GABA degrades is given by *K-_GABA_* and is proportional to the GABA-T activity *(**G_T,act_**)*, where GABA-T is the enzyme that catalyses the degradation reaction:

(3) $$\kappa_{GABA}=\beta_{GABA}GT_{act}$$

The GABA-T activity is inhibited via neuronally derived NO (NO_*n*_) and modelled with a sigmoidal function:

(4) $$GT_{act}=\frac{1}{2}[\left(GT_{\max}+GT_{\min}\right)-\left(GT_{\max}-GT_{\min}\right)\tanh\frac{\left(NO_{n}-GT_{\mathrm{mid}\;\mathrm{point}}\right)}{GT_{\mathrm{slope}}}]$$

where *GT_min_* = 1 is the minimum activity (i.e. when *NO_n_* is at normal resting value, *NO_rest_* ≈ 0.02047 *μ*M and *R_NO_* ≈ 0.02*μ*M), *GT_max_* = 2 is the maximum activity (i.e. when *NO_n_ ≈* 0 *μ*M).

The glutamate concentration can either be increased due to neuronal stimulation as in an excitatory neuron (modelled as a glutamate release when extracellular K^+^concentration *K_e_* is above some threshold) or increased from the degradation of GABA in an inhibitory interneuron (as GABA degrades to succinic semialdehyde and glutamate when catalysed by GABA-T).

### 3.3 Glutamate

The nondimensional glutamate concentration *Glu* is modelled by the following equation:

(5) $$\frac{dGlu}{dt}=f\left(K_{e}\right)+g(GABA)-{\text{β}}_{Glu}Glu$$

where *f (K_e_)* is a function that models the neuronal release of glutamate and f *(GABA)* is a function that models the production of glutamate from the degradation of GABA. These two functions are given by

(6) $$f\left(K_{e}\right)=\frac{{\mathrm{Glu}}_{\max}}{2}\left(1+\tanh\left[\frac{K_{e}-K_{e_{\mathrm{switch}}}}{\mathrm{Glu}u_{\mathrm{slope}}}\right]\right)$$

Here *K_e_switch__* = 5 mM is the concentration above which glutamate is released from the excitatory neuron and *Glu_slope_* = 0.1 mM is the slope of the sigmoidal (both parameters taken from the previous version of the model [23]), and

(7) $$g(GABA)=\kappa_{GABA}GABA$$

where κ_*GABA*_ is the degradation rate of GABA (see equation 3). At rest both *f*(*K_e_*) and *f*(*GABA*) are zero and so the glutamate concentration tends to zero.

The GABA dependent Cl^-^ channel fluxes on the astrocyte and SMC are added to the differential equations for *v_k_* and *v_i_* respectively and are given by

(8) $$J_{GABA,k}=g_{GABA}\left(v_{k}-E_{GABA}\right)$$

(9) $$J_{GABA,i}=g_{GABA}\left(v_{i}-E_{GABA}\right)$$

where v_k_ is the astrocytic membrane potential in mV, *v_i_* is the SMC membrane potential in mV, *E_GABA_* = -75 mV is the reversal potential of the Cl^-^channels, and the GABA dependent conductance of the channels is given by

(10) $$g_{GABA}=\frac{G_{GABA}}{2}\left(1+\tanh\left(\frac{GABA_{N}-g_{\mathrm{mid}}}{g_{\mathrm{slope}}}\right)\right)$$

where *g_mid_* = 0.8 is the midpoint of the sigmoidal, *g_slope_* is the slope of the sigmoidal (both model estimates), and G_GABA_ is the maximal conductance of the channel given by 0. × *G_Cl,i_* where *G_Cl,i_* = 1.34 × 10^−6^ *μ*M mV^−1^ ms^−1^ is the conductance of the SMC Cl^-^leak channel taken from the NVU model. This is a model estimate and can be changed by fitting to data. Hence when *GABA_N_* = 0, *g_GABA_* = 0 and when ^GABA^N = 1, *g_GABA_ = G_GABA_*.

### 3.4 NPY

NPY production is modelled by a time dependent profile which is a function of I(t), the inhibitory neuron output *α_NPY_I_H_* *(t)*. NPY degradation is given by a function of the concentration of NPY, −β_NPY_*(NPY −NPY_base_)*.

Finally the flux of Ca^2+^through the VOCC channel on the SMC is given by

(11) $$J_{VOCC,i}=g_{VOCC}\frac{v_{i}-v_{Ca1}}{1+\exp\left(\frac{-\left(v_{i}-v_{Ca2}\right)}{R_{C}a}\right)}$$

where *v*_*Ca*1_ *=* 100 mV is the reversal potential, *v*_*Ca*2_ = −24 mV is the half-point of the VOCC activation sigmoidal, and *R_Ca_* = 8.5 mV is the maximum slope of the sigmoidal (all taken from the NVU model). The NPY conductance is given by

(12) $$g_{\mathrm{VOCC}}=G_{Ca,i}\left(1+\frac{N_{inc}}{2}\tanh\left(\frac{NPY_{N}-N_{\mathrm{mid}}}{N_{\mathrm{slope}}}\right)\right)$$

where *G_Ca,i_* = 1.29 × 10^−6^ *μ*M mV^−1^ ms^−1^ is the base conductance of the VOCC (taken from the NVU model), *N_inc_* = 0.05 is the proportional increase of the conductance from baseline due to NPY (i.e. 0.05 means a 5% increase from *G_Ca,i_* when NPY is released, model estimate), *N_mid_* = 0.6 is the midpoint of the sigmoidal, and *N_slope_* = 0.1 is the slope of the sigmoidal (both model estimates). Hence when *NPY_N_* = 0, *gvocc = G_ca,i_* and when *NPY_N_* = 1, *gvocc* = 1.05 × *G_ca,i_*.

Equations for the three state variables GABA, NPY and Glu are written as

(13) $$\frac{dGABA}{dt}=-\kappa_{GABA}\left(GABA-GABA_{\mathrm{base}}\right)+\alpha_{GABA}I_{H}(t)$$

(14) $$\frac{dNPY}{dt}=-\beta_{NPY}\left(NPY-NPY_{\mathrm{base}}\right)+\alpha_{NPY}I_{H}(t)$$

(15) $$\frac{dGlu}{dt}=-\beta_{Glu}Glu+\left(f\left(K_{e}\right)+g(GABA)\right)$$

De-oxyhaemoglobin (HbR) is a state variable and is evaluated using the balloon model of Buxton [26], whilst HbO and HbT are calculated using $\text{HbT}=\frac{\text{CBFHbR}}{\text{CMRO}}$ where CBF is the cerebral blood flow in the lumen and CMRO is the metabolic rate of oxygen consumption. HbO is formed from the conservation of haemoglobin such that HbT = HbR+HbO

The full system is written in python using the ‘odeint’ solver and executed on an iMac with 16Gb memory. A single simulation is run using an extended baseline calculation to obtain steady-state values before the neuron is stimulated. The full simulation is complete within 20 seconds.

## 4 Results

### 4.1 Pre-LNAME (‘no drug’) condition

**Fig. 2:**
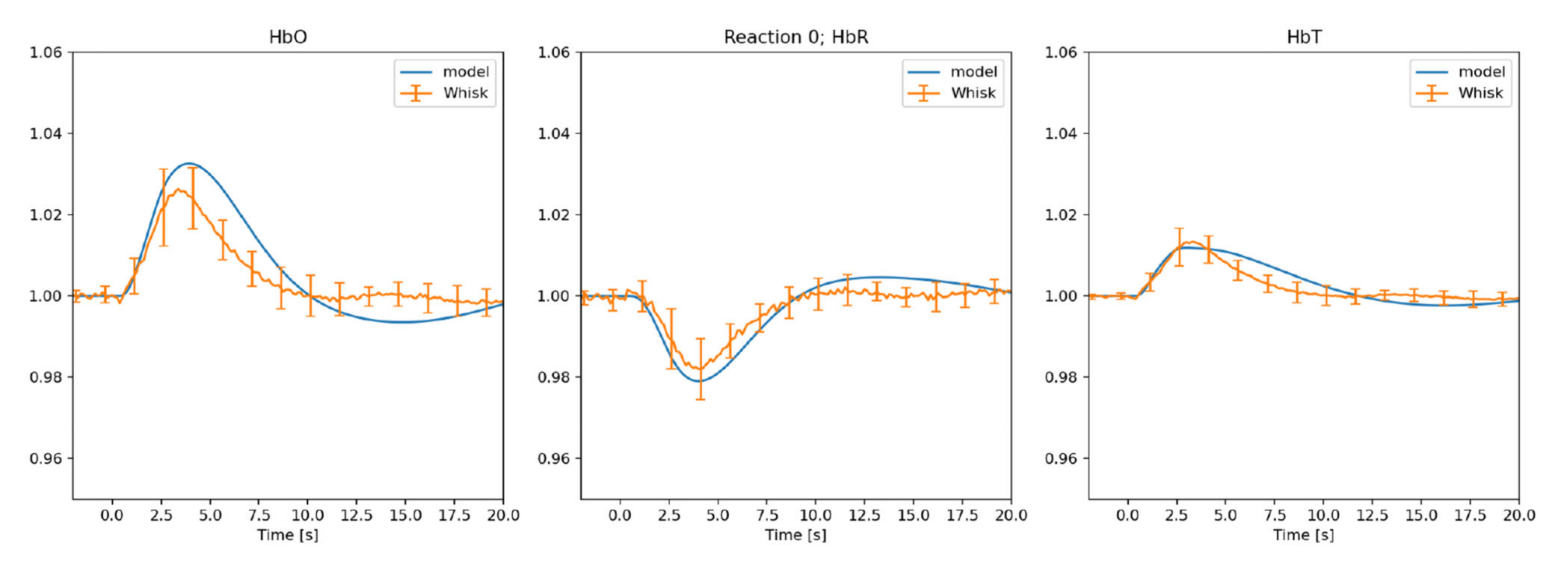
Comparison of experimental data (fractional change of haemoglobin) and numerical model for the case of 2 second whisker stimulation without drug injection.(Orange profile is the mean (n=12) ± s.d. blue profile is the numerical simulation result)

**Fig. 3:**
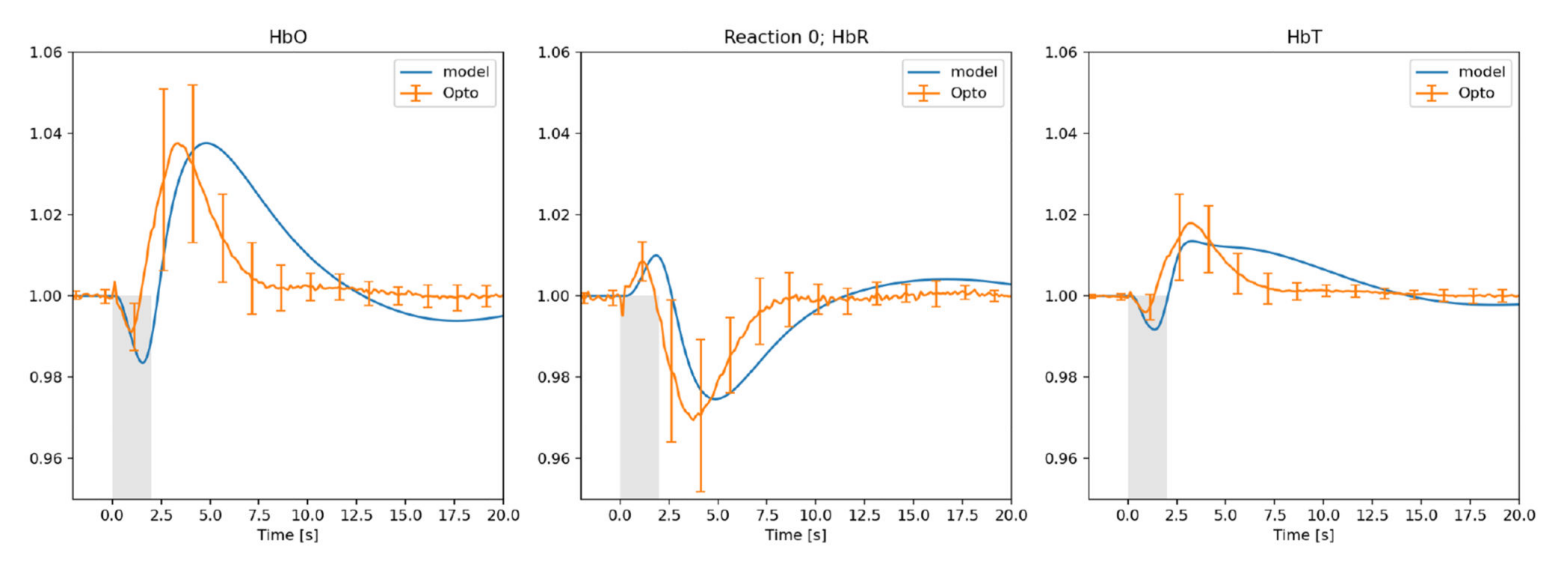
Experimental (fractional change of haemoglobin) and numerical model results for the optogenetic condition before LNAME is introduced. (Orange profile is the mean (n=12) ± s.d. blue profile is the numerical simulation result), grey indicates time period of stimulation.

**Fig. 4:**
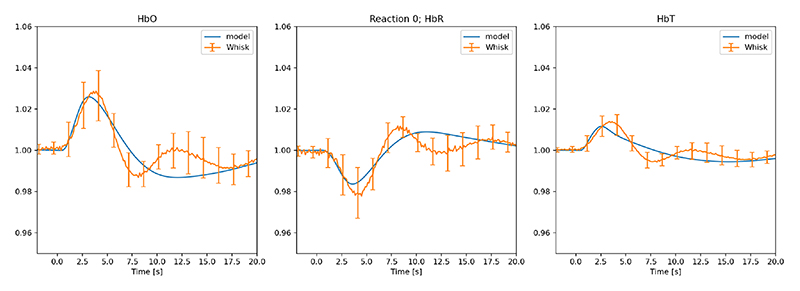
Comparison of experimental data [18] and numerical model for whisker stimulation with LNAME injected

**Fig. 5:**
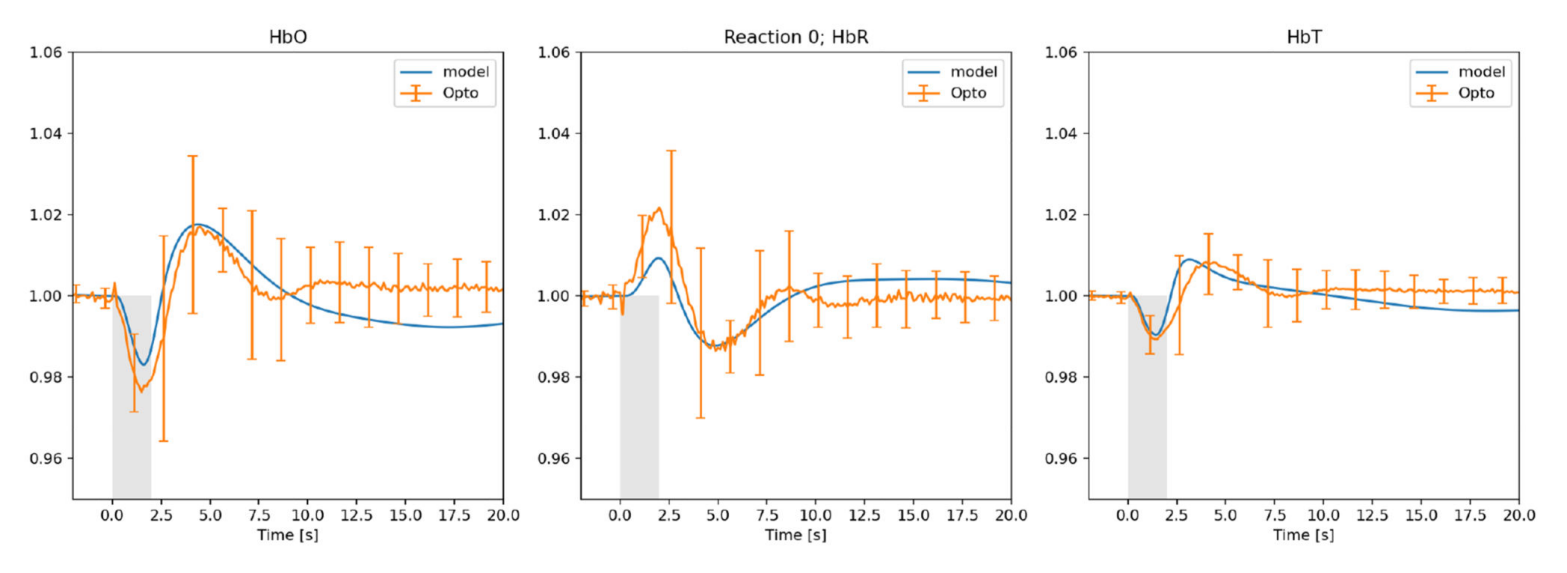
Comparison of experimental data [18] and numerical model results for the optogenetic condition when LNAME is injected, grey indicates time period of stimulation.

The following figures (2-5) showing HbO, HbR and HbT compare the experimental results measured from 12 mice (multiple stimulations per mouse during the experimental procedure) where the orange profile is the mean (n=12) ± s.d. whilst the blue profile is the numerical simulation result. Figure 2 shows the comparison of experimental data [18] and numerical model for the case of 2 sec whisker stimulation before LNAME injection. Figure 3 shows the model results for the optogenetic condition when no drug is introduced. We should note that in order to get the required negative pulse for HbO (and associated positive pulse for HbR at the initial stages of the stimulation) the pulse width for NPY is half that of GABA.

### 4.2 LNAME condition

The experiment using the LNAME drug infusion degrades all NO production. Figure 4 shows the experiment and numerical simulation after LNAME is injected. Figure 5 shows the model results for the optogenetic condition when LNAME is introduced.

## 5 Discussions

We first investigate the whisker stimulation experiments then move on to the optogenetic condition. It should be noted that there is no change in the numerical model parameters between the no drug and LNAME conditions but simply a switch allowing the production of NO to be forced to zero.

### 5.1 Whisker stimulation

Both Figures 2 and 4 show good comparison with experiment. The numerical simulation shown in Figure 2 provides excellent comparison during the first half of the stimulation (t ∈ [0,1] seconds) with HbR and maintains the profile with the ± s.d. until the final 10 seconds when returning to baseline. Initially it was thought that the slower return to baseline from the numerical simulation may have been mediated by 20-HETE (20-Hydroxyeicosatetraenoic acid) however both numerical and experimental [18] evidence indicates otherwise. This is in contrast to the work of Liu et al [27]. The numerical experiments indicate (not shown) that with the production of 20-HETE forced to zero there is no significant difference in the profiles of HbO, HbR or HbT. Figure 4 shows that the numerical model cannot simulate the post-stimulation oscillations. These oscillations occur for both whisker and optogenetic experimental results. It is unclear at present as to how these oscillations come about.

In the case of LNAME infusion the numerical results show good agreement with the experiment although as before the post stimulation oscillations are not reproduced. These results indicate that for the short periods of stimulation NO does not (with somato-sensory (whisker) stimulation) significantly effect neurovascular coupling.

### 5.2 Optogenetic stimulation

In contrast to the whisker stimulation results LNAME does have a substantial effect on GABAergic interneuron stimulation. Figure 3 provides the time profiles for experiment and numerical simulation. Immediately following the start of stimulation HbR shows a small but significant increase with a concomitant decrease in both HbO and HbT. This is due to NPY opening the voltage operated Ca^2+^channel (VOCC) before the GABA concentration has time to open the Cl^-^channels on both the astrocyte and smooth muscle cells. Once NPY has decreased by natural decay the influence of GABA is more prolonged. Reasonably good comparison with experiment, can simulate the initial increase of HbR due to the dominant NPY pathway but the decay for the no-drug condition does not compare as well. Experimental evidence shows that these oscillations are not wholly caused by the influence of 20-HETE [18].

For the LNAME optogenetic case the numerical results show reasonable agreement although the comparison with HbR is not as good in the initial stages as in the whisker case. For all three haemoglobin profiles the simulation says within ± s.d.. The experimental and numerical profiles show a substantial difference between no drug and LNAME conditions. By looking at Figure 1 it is seen how this difference may occur. With NO present enzyme GABA-T (GABA transaminase) inhibits the degradation of GABA to glutamate allowing GABA to open the Cl^-^channel resulting in the smooth muscle cell hyperpolarising thence closing the VOCC channel with the consequence of vasodilation. With NO absent GABA now degrades to glutamate allowing the NPY pathway to dominate; essentially a balance of pathways mediated by NO.

## 6 Conclusions

Models of the stimulation of GABA-ergic interneurons with the inclusion of both GABA and NPY concentrations has been integrated into the numerical model of [13,1]. Time profiles of HbO, HbR and HbT are generated from the model and compared with the experimental data of [18]. The model supports the conclusion of Lee et al [18] that NO is not responsible for initial sensory-induced neurovascular coupling within the mouse cortex. The model results also show that with the optogenetic stimulation of nNOS-expressing interneurons GABA and NPY play a ‘balancing’ role mediated by NO in reducing the inhibition of GABA-T thus allowing a substantial increase in Glutamate within the extracellular space. Further work will need to be done to investigate the role of NO when stimulation periods are increased substantially from the short pulses of 2 seconds as used in the above experiments.

## Figures and Tables

**Table 1:** New parameters of the GABA and NPY model.

Parameter	Description	Value	Refe
*G_T,min_*	Minimum GABA-T activity	1	[22]
*G_T,max_*	Maximum GABA-T activity	2	[22]
*E_GABA_*	Reversal potential of the GABA dependent Cl^-^channels	−75 mV	[24]
*G_GABA_*	Conductance of GABA dependent CT^-^channel	4.02 × 10^−7^ *μ*M mV^−1^ ms^−1^	[25]
*α_GABA_*	non-dimensional production rate of GABA	1.6 × 10^−3^	M.E.
β_GABA_	GABA activity rate	2.2 × 10^−3^	M.E.
*α_NPY_*	non-dimensional production rate of NPY	4.2 × 10^−3^	M.E.
*β_NPY_*	NPY degradation rate	2.2 × 10^−3^	M.E.
*β_Glu_*	Glutamate degradation rate	4.2 × 10^−3^	M.E.
*GT_mid point_*	value of NO at which 1/2 maximal	0.06	M.E.
*GT_slope_*	sigmoidal rate	0.02128	M.E.
*g_mid_*	sigmoidal of Cl^-^channel	0.5	M.E.
*g_slope_*	sigmoidal of Cl^-^channel	0.15	M.E.
*N_inc_*	proportional/percentage increase of the conductance	*x*	M.E.
*N_mid_*	1/2 maximal NPY value for VOCC	0.5	M.E.
*N_slope_*	sigmoidal rate for VOCC	0.15	M.E.
